# Glipizide suppresses prostate cancer progression in the TRAMP model by inhibiting angiogenesis

**DOI:** 10.1038/srep27819

**Published:** 2016-06-13

**Authors:** Cuiling Qi, Bin Li, Yang Yang, Yongxia Yang, Jialin Li, Qin Zhou, Yinxin Wen, Cuiling Zeng, Lingyun Zheng, Qianqian Zhang, Jiangchao Li, Xiaodong He, Jia Zhou, Chunkui Shao, Lijing Wang

**Affiliations:** 1 Vascular Biology Research Institute, School of Basic Course, Guangdong Pharmaceutical University, Guangzhou 510006, China; 2 Department of Pathology, the Third Affiliated Hospital, Sun Yat-sen University, Guangzhou, Guangdong 510630, China; 3 Chemical Biology Program, Department of Pharmacology and Toxicology, University of Texas Medical Branch, Galveston, TX 77555, United States

## Abstract

Drug repurposing of non-cancer drugs represents an attractive approach to develop new cancer therapy. Using the TRAMP transgenic mouse model, glipizide, a widely used drug for type 2 diabetes mellitus, has been identified to suppress prostate cancer (PC) growth and metastasis. Angiogenesis is intimately associated with various human cancer developments. Intriguingly, glipizide significantly reduces microvessel density in PC tumor tissues, while not inhibiting prostate cancer cell proliferation from the MTT assay and flow cytometry investigation. Moreover, glipizide inhibits the tubular structure formation of human umbilical vein endothelial cells by regulating the HMGIY/Angiopoietin-1 signaling pathway. Taken together, these results demonstrate that glipizide has the potential to be repurposed as an effective therapeutic for the treatment of PC by targeting tumor-induced angiogenesis.

Prostate cancer (PC) is the most frequently diagnosed malignant tumor in men with a rising incidence, and is one of leading causes of cancer-related deaths^1^,^2^. PC proceeds via multiple steps including initiation, promotion, and progression^3^. During its initial stages, the prognosis of patients with PC is relatively satisfactory after receiving surgical resection and/or radiation therapy. Unfortunately, at an advanced stage, patient outcomes even with treatment are quite poor^4^. Despite the high prevalence of PC, very few drugs are available for inhibiting PC progression. Thus, there is an urgent need to identify effective agents capable of significantly suppressing prostate carcinogenesis.

Glipizide is an antidiabetic drug that has been used for Type II diabetes mellitus (T2DM) treatment since the 1950s by stimulating insulin secretion from β-cells^5^. The incidence of cancer in T2DM patients has been shown to be higher than in the general population^6^. Excitingly, recent pharmacoepidemiological surveys have shown that long-term use of anti-diabetic drugs may be associated with reduced cancer risk in T2DM patients. For example, the use of gliclazide and glibenclamide, close analogs of glipizide, may result in reduced cancer risk in a dose-dependent manner^6^. Interestingly, we screened an FDA-approved drug library and found that glipizide significantly inhibited blood vessel formation^7^. Further studies also demonstrated that glipizide inhibited breast cancer growth and metastasis in 4T1 transplanted tumors and spontaneous breast cancer in MMTV-PyMT transgenic mice by inhibiting angiogenesis through up-regulating natriuretic peptide receptors A^7^. However, whether glipizide suppresses PC tumor growth and metastasis has never been explored, and its role in inhibiting prostate cancer progression remains unclear.

Angiopoietin-1 (ANGPT1), produced by pericytes and vasculature support cells, is a member of the angiopoietin family that includes angiopoietin-1, angiopoietin-2 and angiopoietin-4 8. It has been reported that ANGPT1 is required to maintain the stability of mature vessels through pericyte recruitment and formation of non-leaky vessels^9^. ANGPT1 also plays an important role in maintaining and stabilizing the tumor vasculature. Not only mural cells and fibroblasts but also non-vascular normal and tumor cells, including colorectal carcinoma^10^, bladder cancer^11^ and PC^12^, express ANGPT1. Studies have shown that increased expression of ANGPT2 relative to ANGPT1 in tumors is related to a poor prognosis for a variety of cancers, and ANGPT1 plays an essential role in tumor angiogenesis^13^,^14^. Furthermore, there are high-mobility group I-Y (HMGIY) binding sites in ANGPT1 promoters and HMGIY can control ANGPT1 expression in endothelial cells^15^. HMGIY protein belongs to family A, which contains three basic DNA-binding domains (the so-called AT-hooks) and includes four member coded by HMGIY and high-mobility group I-C (HMGIC) genes^16^. In addition, HMGIY is abnormally overexpressed in many malignant tumors such as cervical, colon, prostate or thyriod cancers and closely related to tumor progression^17^,^18^,^19^. However, it remains obscure whether glipizide inhibits angiogenesis by regulating the HMGIY/ANGPT1 signaling pathway.

In the present study, to assess the suppressive effects of glipizide on PC, we used transgenic TRAMP mice, which spontaneously develop adenocarcinoma of the prostate^20^. The TRAMP mouse model is based on SV40 large T antigen (T Ag) expression in the prostate epithelium^21^. T Ag expression effectively abrogates the p53 protein and retinoblastoma functions and serves as an oncoprotein. As a result, TRAMP mice spontaneously develop prostatic adenocarcinoma from severe hyperplasia at 12 weeks of age due to poorly differentiated and invasive adenocarcinomas at 24 weeks of age, which finally become distant metastasis^22^,^23^,^24^. Furthermore, to determine the effect of glipizide on PC, TRAMP mice with a C57BL/6 background were crossed with FVB mice to obtain the [C57BL/6 × FVB]F1 TRAMP mice. The [C57BL/6 × FVB]F1 TRAMP mice develop a more rapidly progressing PC and have more abundant blood vessels than TRAMP mice with a C57BL/6 background. Compared with the C57BL/6 TRAMP mice, the [C57BL/6 × FVB]F1 TRAMP mice are more useful to study tumor angiogenesis.

Repurposing of non-cancer drugs represents an attractive approach for developing new cancer therapy, given that it may significantly reduce the investigational time and cost from bench side to bedside. Herein, we report the effects of glipizide on PC using TRAMP mice via injecting the drug into the abdominal cavity, indicating that glipizide remarkably suppresses PC progression. Further studies have revealed that glipizide suppresses tumor-induced angiogenesis through down-regulating HMGIY/ANGPT1 signals, but not tumor cell proliferation, thereby inhibiting PC tumor growth and metastasis. These findings suggest that glipizide has the potential to be repurposed as an effective therapy that may benefit PC patients.

## Results

### Glipizide suppresses prostate cancer growth in TRAMP mice

**Figure 1:**
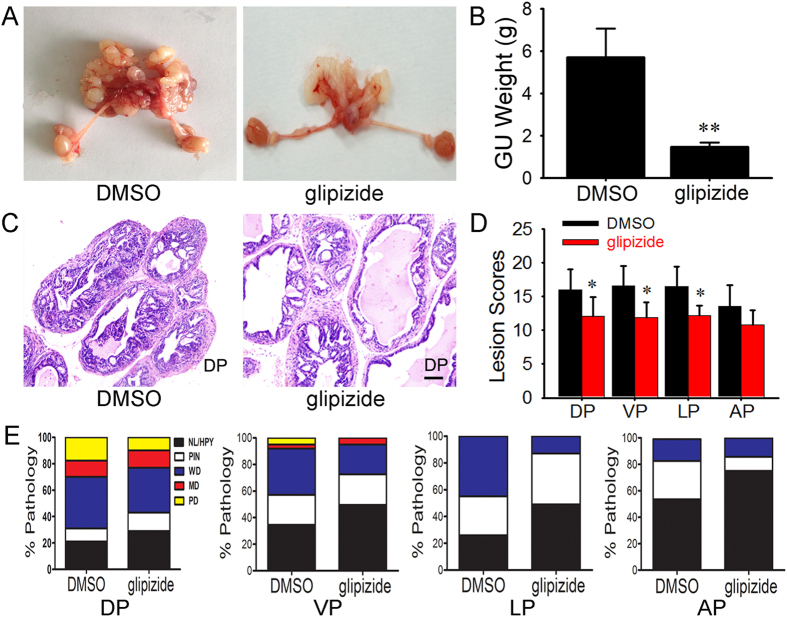
Glipizide suppresses tumor growth in spontaneous PC. Male [C57BL/6 × FVB]F1 TRAMP mice (16 weeks of age) were allowed to spontaneously develop PC and injected with glipizide (5 mg/kg) or DMSO (control) once every three days for 8 weeks. After the mice were sacrificed, the GU tracts were harvested and weighed. (**A**) The images show the representative gross appearance of the GU tracts from TRAMP mice treated with glipizide and DMSO. (**B**) There is a significant difference between the relative GU weights of glipizide- or DMSO-treated tumors. (**C**) Representative H&E images of DP tissues from TRAMP mice treated with DMSO and glipizide. (**D**) The lesion scoring in every prostate lobe of DMSO- and glipizide- treated TRAMP mice. (**E**) The lesion distribution in the individual prostate lobes of DMSO- and glipizide-treated TRAMP mice. *p < *0.05;* **p < *0.01*.

To determine whether glipizide inhibits PC growth, we employed a spontaneous murine model of prostate adenocarcinoma. Glipizide (5 mg/kg) was injected into 16-week-old TRAMP mice with a [C57BL/6 × FVB]F1 background every three days. The weights of the genitourinary (GU) tract were measured following 8 weeks of glipizide or control treatment. The relative GU tract weights of glipizide-treated TRAMP mice were significantly less than those of control mice (Fig. 1A,B).

Given that glipizide is capable of inhibiting the relative GU weights of TRAMP mice, we speculated that glipizide may also affect the lesion score and pathological grade. To this end, we evaluated the pathological characteristics of the two groups through a TRAMP-specific grading score and pathological classifications. The representative images of hematoxylin and eosin (H&E) staining displayed the morphological differences between glipizide- and DMSO-treated mice (Fig. 1C, Supplemental Fig. S1). At a morphological level, glipizide reduced lesion severity, and according to the TRAMP-specific grading scheme^25^, there was a significant reduction in the lesion scores of dorsal prostate (DP), ventral prostate (VP) and lateral prostate (LP) lobes of the TRAMP mice treated with glipizide. Interestingly, glipizide did not affect the lesion scores of anterior (AP) prostate lobes of the TRAMP mice (Fig. 1D). As previously described, the prostates of TRAMP mice were classified as normal/hyperplasia (NL/Hyp), prostatic intraepithelial neoplasia (PIN), well-differentiated (WD) adenocarcinoma, moderately differentiated (MD) adenocarcinoma, or poorly differentiated (PD) adenocarcinoma^26^. Compared with the TRAMP mice treated with DMSO, the TRAMP mice treated with glipizide in the overall pathology of the prostate have shown a higher percentage of NL/Hyp tissues (75% vs. 53.6% in AP, 29% vs. 21% in DP, 49.5% vs. 34.6% in VP, 49% vs. 26% in LP; Fig. 1E). Furthermore, TRAMP mice treated with glipizide also displayed a higher percentage of PIN tissues (29% vs. 10.83% in AP, 14% vs. 10% in DP, 23% vs. 22.4% in VP, 38% vs. 29% in LP) (Fig. 1E). However, there was a lower percentage of prostate tissues in the PC classifications of the TRAMP mice treated with glipizide than those of the TRAMP mice treated with DMSO as observed for WD adenocarcinoma (12.3% vs. 13% in DP, 3% vs. 5% in VP) and PD adenocarcinoma (10% vs. 17.7% in DP, 0% vs. 5% in VP; Fig. 1E). There were no MD or PD tissues in the TRAMP mice treated with both DMSO and glipizide.

### Glipizide inhibits prostate cancer metastasis in TRAMP mice

**Figure 2:**
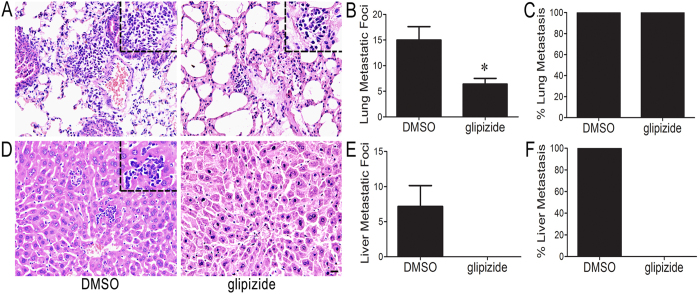
Glipizide inhibits PC metastasis. (**A**) Representative H&E images of lung tissues show that there are metastatic tumors in the lungs of the TRAMP mice treated with glipizide and DMSO. Higher magnifications of the metastatic tumors are shown in the black dotted squares. (**B**) The glipizide-treated TRAMP mice develop fewer metastatic foci than the DMSO-treated mice. (**C**) All TRAMP mice treated with glipizide or DMSO develop lung metastases of PC. (**D**) H&E staining of liver tissues sections demonstrates that there are liver metastatic foci of PC in the DMSO-treated TRAMP mice but not in the glipizide-treated TRAMP mice. Higher magnifications of the metastatic tumors are shown in the black dotted squares. (**E**) The liver metastasis accumulating in the TRAMP mice after glipizide and DMSO treatment. (**F**) Incidence of glipizide- or DMSO-treated TRAMP mice with liver metastasis. Bar, 20 μm. *p < *0.05*.

Given that glipizide is capable of inhibiting PC growth, glipizide may have an important role in PC metastasis. As expected, glipizide significantly suppressed lung and liver metastasis compared with the DMSO group. Metastatic foci were not found in other organs such as kidneys, brains and spleens. The numbers of lung and liver metastatic foci in the TRAMP mice treated with glipizide were fewer than those in the mice treated with DMSO (Fig. 2A,B,D,E). Furthermore, all the TRAMP mice treated with either DMSO or glipizide were found to develop lung metastasis (Fig. 2C). While all the TRAMP mice treated with DMSO developed liver metastasis, the glipizide-treated TRAMP mice did not (Fig. 2F). Meanwhile, glipizide significantly decreased the postprandial blood glucose levels of the TRAMP mice, but did not affect the body weights and blood lipid levels of the TRAMP mice (Supplemental Table S1 and Supplemental Fig. S3). Immunohistochemical staining with T-antigen antibodies was performed to determine whether glipizide affected transgene expression. The results demonstrated that glipizide had no effect on transgene expression (Supplemental Fig. S4). The findings suggested that glipizide inhibited the lung and liver metastasis of PC in TRAMP mice.

### Glipizide suppresses tumor-induced angiogenesis, but does not affect prostate cancer cell proliferation

**Figure 3:**
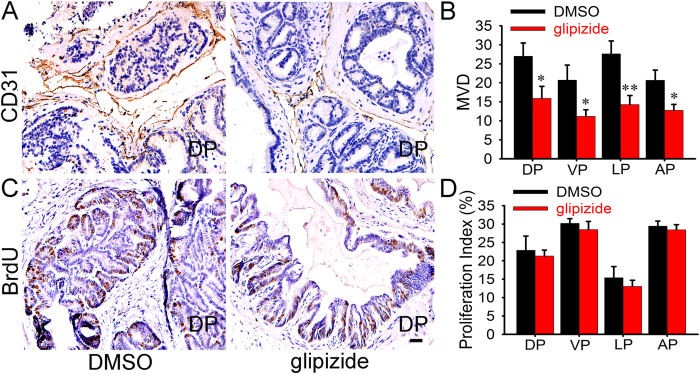
Glipizide inhibits tumor-induced angiogenesis, but does not affect cell proliferation in spontaneous PC. (**A**,**B**) Immunohistological staining for blood vessel with CD31 was performed in the DP, VP, LP and AP lobes of the prostates. Compared with the DMSO group, the staining indicates the microvascular density is significantly decreased in the glipizide-treated mice. (**C**,**D**) Immunohistological staining against BrdU was performed on DP, VP, LP and AP lobes of the prostates. The staining indicates that glipizide exhibits no significant effect against PC cell proliferation. Bar, 50 μm. *p < *0.05*, **p < *0.01*.

In view of our previous report that glipizide exhibited no effect on breast cancer cell proliferation while suppressing breast cancer growth and metastasis through inhibiting angiogenesis^7^, we investigated the role of glipizide in angiogenesis in PC. Immunohistochemical staining (IHC) for CD31 was performed on PC sections. Compared with the control, the microvessel density in DP, VP, LP and AP of glipizide-treated prostate tumors was significantly reduced (Fig. 3A,B, Supplemental Fig. S2). These results suggested that glipizide inhibited PC growth by impeding tumor-induced angiogenesis *in vivo*.

After showing that glipizide can inhibit PC growth in TRAMP mice, it deems necessary to explore whether glipizide can inhibit PC cell proliferation. Immunohistological staining for BrdU was performed to determine the effect of glipizide against tumor cell proliferation. These results demonstrate that glipizide has no significant effect on PC cell proliferation (Fig. 3C,D, Supplemental Fig. S2).

### Glipizide inhibits microvessel formation

**Figure 4:**
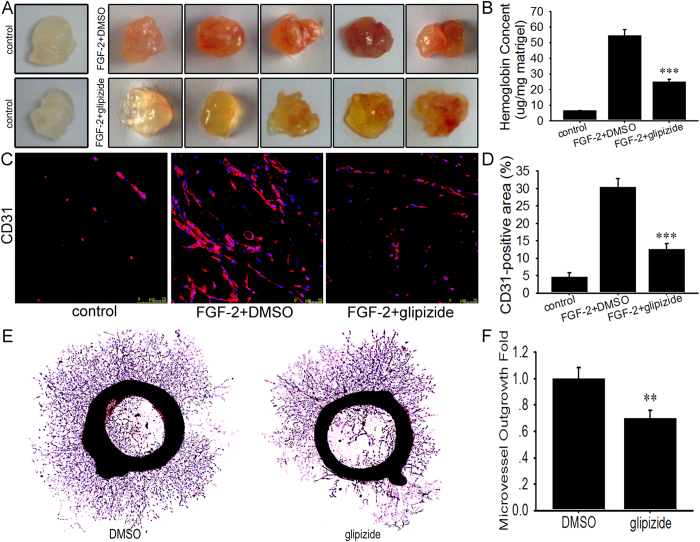
The effects of glipizide on Matrigel plug vascularization and the angiogenic sprouting of the aortic rings. Matrigel was mixed with either DMSO and FGF-2, or glipizide and FGF-2, and injected subcutaneously into Balb/c mice. After 7 days, Matrigel plugs were isolated and removed, and then the images of Matrigel plugs were taken. (**A**) Representative images of control (without glipizide and FGF-2), DMSO containing FGF-2, glipizide containing FGF-2. (**B**) The total amounts of hemoglobin in the plugs. The plugs treated with DMSO and glipizide were lysed and the levels of hemoglobin were determined by ELISA. (**C**) The plugs were excised, followed by immunostaining for CD31 antibodies. (**D**) The percentage of CD31-positive area. (**E**) The aorta was isolated from the Sprague-Dawley rats and was cut into 1–2 mm long rings. The rings were placed into Matrigel-coated plate and treated with DMSO or glipizide. Representative photograghs of sprouts from aortic rings. (**F**) Microvessel outgrowth was quantified. **p < *0.01*, ***p < *0.001*.

We have previously reported glipizide could inhibit angiogenesis on the vascular plexus of CAM and YSM^7^. To further determine the antiangiogenic properties of glipizide, Matrigel plugs and rat aortic ring assays were performed. For the *in vivo* Matrigel plug angiogenesis assays, Matrigels mixed with FGF-2, heparin or glipizide, were injected subcutaneously into Balb/c mice. Seven days later, Matrigel plugs were removed. As shown in Fig. 4A, the plugs of control group (without FGF-2 and glipizide) appeared white. Matrigel plugs containing FGF-2 and DMSO appeared deep red, demonstrating that functional vessels had formed in the Matrigel plugs. However, the plugs containing glipizide (25 μg per plug) and FGF-2 dramatically showed light red, indicating that very few blood vessels have formed (Fig. 4A). We next determined the amount of hemoglobin in the plugs. As expected, the amounts of hemoglobin from the plugs treated with glipizide were significantly lower than those from plugs with DMSO (Fig. 4B). Furthermore, immunofluorescent staining of the functional blood vessels in Matrigel plugs indicated that glipizide inhibited angiogenesis (Fig. 4C,D).

In addition to the *in vivo* Matrigel plug assay, the *ex vivo* rat aortic ring assay was performed. In *ex vivo* rat aortic ring, glipizide significantly inhibited the sprouting of microvessels as evidenced by a decrease in the percentage of microvessel outgrowth (Fig. 4E,F).

### Glipizide does not affect PC-3, 22Rv1 and DU145 prostate cancer cell proliferation

**Figure 5:**
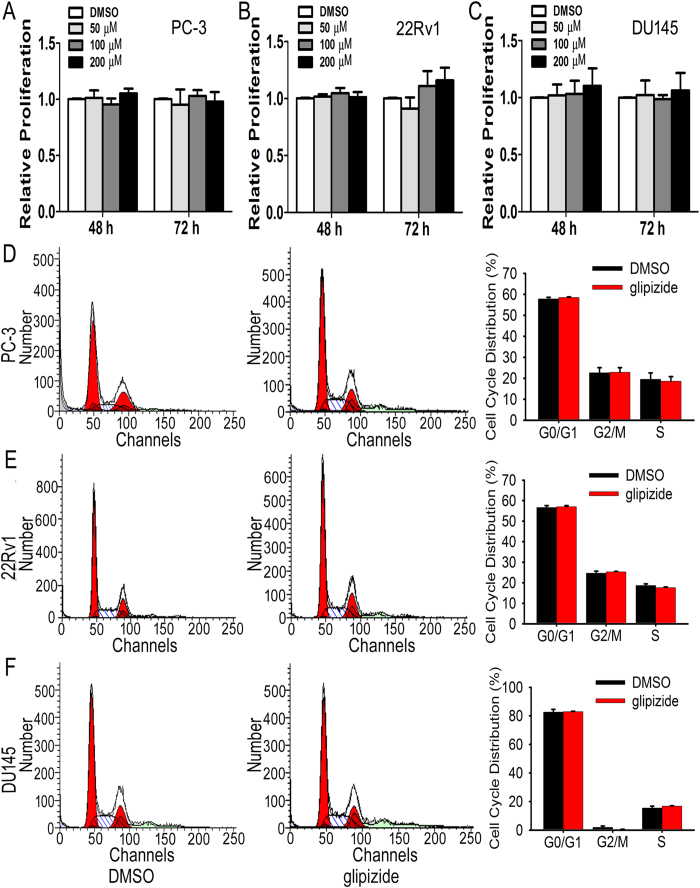
Glipizide displays no role in PC cell proliferation. (**A**–**C**) PC cells, including PC-3, 22Rv1 and DU145 cells, were treated with 50–200 μM glipizide for 48 h or 72 h. Next, an MTT assay was performed. The results indicate no effect on cell proliferation and viability. (**D**–**F**) PC-3, 22Rv1 and DU145 cells were treated with 200 μM glipizide for 48 h, and the PC cell cycle was analyzed by flow cytometry. The results from flow cytometry show that glipizide does not influence the cell cycle distribution of PC-3, 22Rv1 or DU145 cells.

To study whether glipizide directly inhibits PC cell proliferation *in vitro*, we investigated the effects of glipizide against PC-3, 22Rv1 and DU145 PC cell proliferation using an MTT assay. After these cells were treated with 50–200 μM glipizide for 48 h or 72 h, an MTT assay was performed to detect cell viability. It was found that glipizide exhibited no significant effects on PC cell proliferation at all tested concentrations varying from 50 μM to 200 μM (Fig. 5A–C). To further explore the effect of glipizide on PC cell proliferation, we treated PC cells with 200 μM glipizide for 48 h and detected the cell cycle distribution using flow cytometry. Cell cycle analysis by flow cytometry demonstrated that glipizide did not substantially affect PC cell proliferation (Fig. 5D–F).

### Glipizide inhibits angiogenesis through the down-regulation of vascular ANGPT1 expression

**Figure 6:**
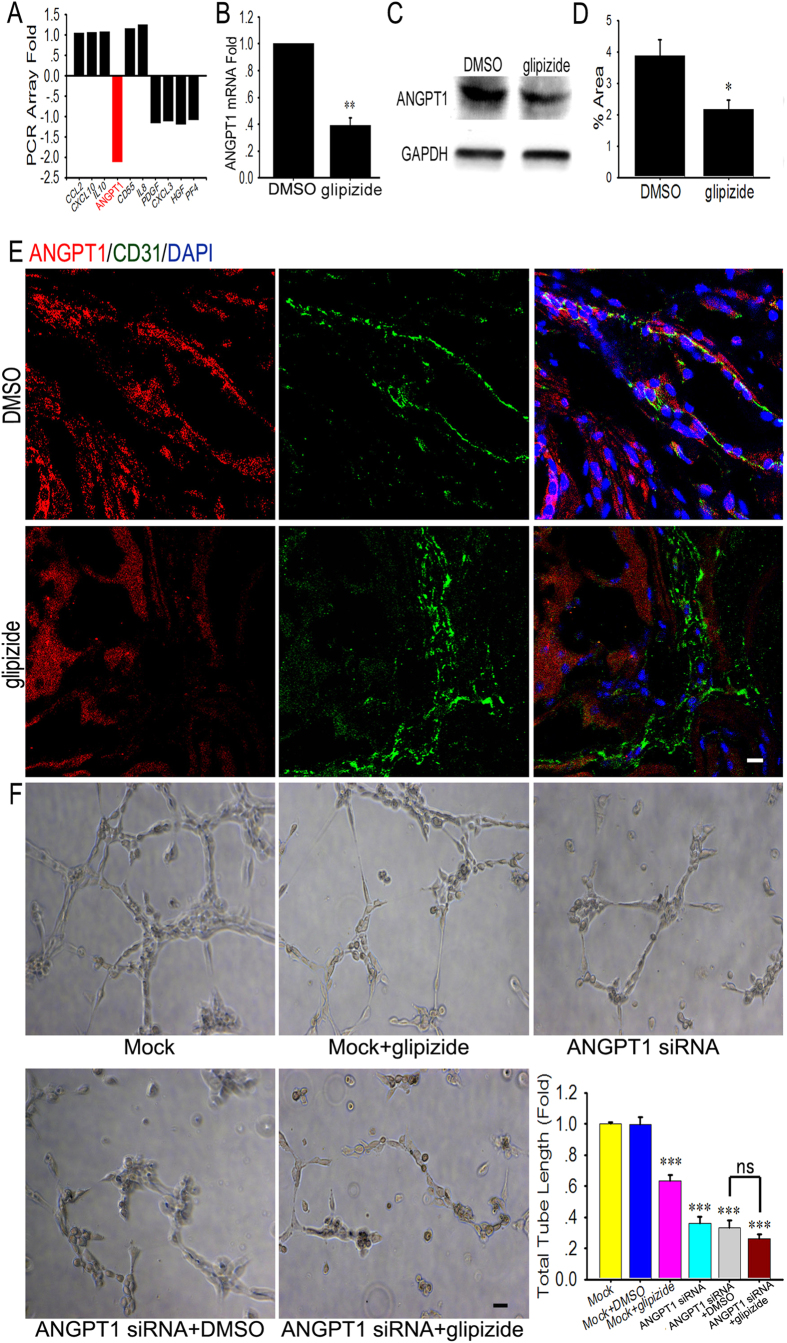
Glipizide inhibits angiogenesis through down-regulation of ANGPT1 expression. (**A**) ANGPT1 expression is down-regulated in HUVECs treated with glipizide by qRT-PCR array analysis. (**B**,**C**) To further determine the qRT-PCR array results, qRT-PCR (**B**) and Western blotting (**C**) were performed. (**D**,**E**) Immunofluorescent staining shows that the levels of ANGPT1 expression are high in the blood vessels of tumor tissues of TRAMP mice treated with DMSO. However, ANGPT1 expression is significantly decreased in the blood vessels of tumor tissues of TRAMP mice treated with glipizide. (**F**) ANGPT1 silencing and glipizide significantly inhibit HUVEC tube formation. In addition, glipizide does not affect the ability of HUVECs to form tubular structures after ANGPT1 is silenced. Scale bars, 20 μm in E and 50 μm in F. *p < *0.05*, **p < *0.01*, ***p < *0.001*.

A qRT-PCR array was performed to analyze angiogenesis-associated genes, and ANGPT1 expression was found to be significantly down-regulated (Fig. 6A). To further determine whether ANGPT1 expression was down-regulated in HUVECs treated with glipizide, qRT-PCR and western blotting were performed. We found that ANGPT1 expression was decreased in HUVECs treated with glipizide (Fig. 6B,C). Next, we detected ANGPT1 expression in the blood vessels of TRAMP mice treated with glipizide and DMSO. We found that, compared with TRAMP mice treated with DMSO, ANGPT1 expression was down-regulated in the blood vessels of TRAMP mice treated with glipizide (Fig. 6D,E). Furthermore, in *ex vivo* rat aortic ring, ANGPT1 significantly induced microvessel sprouting and formed a vessel network around the aortic rings after incubation for 7 days (Supplemental Fig. S5A). Glipizide significantly antagonized the ANGPT1-stimulated sprouting (Supplemental Fig. 5B). We also evaluated the significance of ANGPT1 in angiogenesis by investigating whether silencing ANGPT1 can inhibit HUVEC tube formation. The HUVEC tube formation was decreased and not affected by glipizide after ANGPT1 was silenced (Fig. 6F).

### HMGIY/ANGPT1 signaling pathway is a dominant target of glipizide

**Figure 7:**
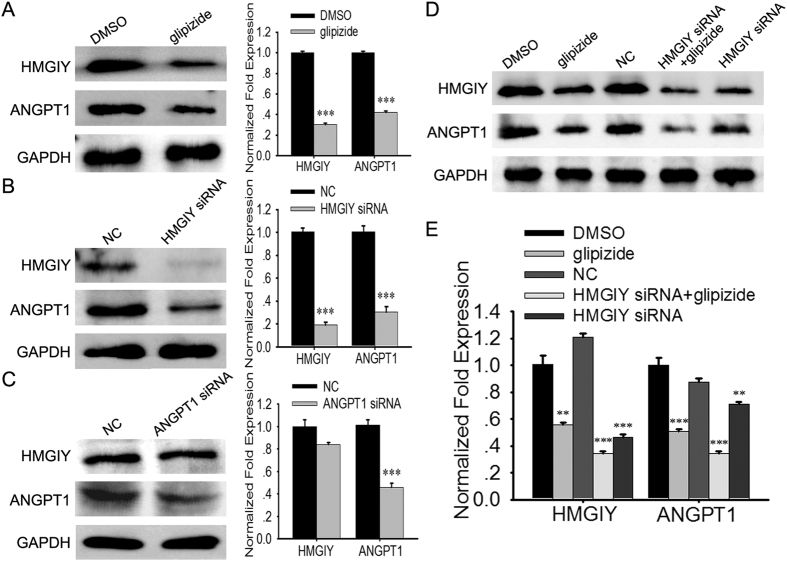
Glipizide targets HMGIY/ANGPT1 signaling pathway in HUVECs. (**A**) Glipizide significantly suppresses the protein expression of HMGIY and ANGPT1. (**B**) Silencing HMGIY inhibits the protein expression of ANGPT-1. (**C**) ANGPT1 deficiency does not affect the expression of HMGIY. (**D,E**) Glipizide does not change HMGIY and ANGPT1 expression in silenced HUVECs. **p < *0.01*, ***p < *0.001*.

We have demonstrated that glipizide can inhibit angiogenesis through down-regulating ANGPT-1 expression. Previous studies have shown that HMGIY can control ANGPT1 expression in endothelial cells^15^. To further explore how glipizide inhibits ANGPT1, we firstly investigated the expression of HMGIY and ANGPT1 in the HUVECs treated with DMSO or glipizide. As shown in Fig. 7A, glipizide suppressed the HMGIY and ANGPT1 protein expression in HUVECs. As expected, silencing HMGIY suppressed ANGPT1 expression, but HMGIY expression did not change after ANGPT1 was silenced (Fig. 7B,C). Further research also showed that glipizide did not affect HMGIY and ANGPT1 expression after HMGIY was silenced (Fig. 7D,E). These results indicate that HMGIY and ANGPT1 may be one of the predominant targets of glipizide.

To further determine the up-stream regulated signaling molecules of ANGPT1, we investigated whether glipizide has an effect in HUVEC cells with a constitutively activated ANGPT1-receptor. It was found that glipizide significantly up-regulated the Tie2 and p-Tie2 protein expression in HUVECs (Supplemental Fig. S5C,D). We also explored whether glipizide had an effect on tube formation of HMGIY-silenced HUVECs stimulated with exogenous ANGPT1. The results demonstrated that the HUVEC tube formation was not affected by exogenous ANGPT1 after HMGIY was silenced (Supplemental Fig. S5E,F). These findings supported that HMGIY was the up-stream signal of ANGPT1. Furthermore, the ^1^H NMR was performed to investigate whether glipizide binds to HMGIY or ANGPT1 proteins. These results demonstrate that glipizide dose not directly bind to either HMGIY or ANGPT1 proteins (Supplemental Fig. S6), indicating that HMGIY is not the direct target of glipizide.

## Discussion

In this study, we report the important finding that glipizide, an antidiabetic drug widely used for T2DM, significantly suppresses PC tumor growth and metastasis by inhibiting tumor-induced angiogenesis. Intriguingly, glipizide reduces the microvessel density in spontaneous PC TRAMP mice, thereby preventing PC growth and metastasis.

PC is one of the most diagnosed cancers in the United States, and the incidence is even higher in the United States than that in China, with a rapidly increasing trend^27^,^28^. Despite the high incidence of PC in men worldwide, very few drugs are available for an effective treatment. Epidemiological investigations have indicated that the use of some antidiabetic drugs is associated with a lower cancer risk in T2DM^6^. It was also reported that glipizide in combination with metformin is related to a lower cancer incidence^6^. The growth- and metastasis-inhibiting effects of metformin in liver, lung, breast, gastric, and colorectal cancers have been reported^29^,^30^. The observational studies and clinical trial evidence have demonstrated that metformin use is closely associated with a reduction in the risk of developing liver, colorectal, pancreatic and colorectal cancers^31^. Nevertheless, the role of glipizide in cancer progression has never been specifically explored until such effort is made from our team. We have previously documented that glipizide suppresses breast cancer growth and metastasis using xenograft mouse models with breast carcinoma 4T1 cells and spontaneous breast carcinoma MMTV-PyMT mice^7^. However, whether glipizide can inhibit PC growth and metastasis remains unknown. Our results reveal that glipizide significantly inhibits PC growth and metastasis in TRAMP mouse models, in which mice spontaneously develop adenocarcinoma of the prostate^21^. These findings support that glipizide has the potential to be repurposed as an effective therapeutic agent for the treatment of PC. Furthermore, our previous studies have demonstrated that the inhibition of tumor growth induced by glipizide treatment was primarily attributed to attenuate angiogenesis instead of hypoglycemia^7^.

It has also been reported by our group that glipizide suppresses blood vessel formation and development in embryo chorioallantoic membrane and yolk sac membrane models^7^. Furthermore, glipizide inhibits breast cancer growth and metastasis through suppressing tumor-induced angiogenesis^7^. In addition, glipizide inhibits the microvessel formation in *in vivo* Matrigel plug assay and the *ex vivo* rat aortic ring assay. Moreover, immunohistochemistry with CD31 antibodies was performed to determine whether glipizide inhibits PC growth and metastasis through restraining tumor-induced angiogenesis. The IHC results indicate that glipizide suppresses tumor-induced angiogenesis, thereby affecting PC growth and metastasis. Our MTT assay and flow cytometry study suggest that glipizide does not significantly affect PC cell proliferation. It is known that angiogenesis is a necessary step for tumor growth and metastasis. In addition to antidiabetic activities, the anti-angiogenic properties of glipizide may be applied for PC treatment. One of such successful examples is thalidomide, which displays multiple functions as an anti-angiogenic drug^32^. Thalidomide was initially used to treat morning sickness, a practice that it was later stopped as it led to abnormalities in infants. However, thalidomide is now repositioned for cancer treatment because of its excellent anti-angiogenic effects.

To investigate how glipizide inhibits angiogenesis, we performed qRT-PCR analysis using glipizide-treated HUVECs for angiogenesis-related genes. We have determined that glipizide suppresses angiogenesis through the down-regulation of ANGPT1 expression in vascular endothelial cells. It has been reported that ANGPT1 expression is up-regulated in various types of cancers, such as glioma and plasma cell tumors^33^,^34^. Furthermore, the up-regulated expression of ANGPT1 in many tumors promotes tumor growth. It has recently been demonstrated that ANGPT1 enlarges blood vessels and promotes the recruitment of mural cells during anti-VEGF-A therapy, thereby limiting tumor hypoxia^35^. Our experiments also support that glipizide down-regulates ANGPT1 expression in HUVECs and can not affect tube formation when ANGPT1 is silenced (Fig. 6). Furthermore, glipizide significantly suppresses the ANGPT1-stimulated sprouting in *ex vivo* rat aortic ring (Supplemental Fig. 5). It has been shown that HMGIY can be a regulator of the angiogenic process^15^. In addition, ANGPT1/HMGIY signaling is involved in endothelial cell survival in rat brain microvascular endothelial cells and promotes cerebral ischemia^15^. Overexpression of HMGIY can promote tumor growth and metastasis^29^. Moreover, the HMGIY/ANGPT1 signaling pathway plays an important role in tumor angiogenesis. Hence, the blockade of HMGIY/ANGPT1signaling by glipizide presents an effective therapeutic strategy for inhibiting tumor angiogenesis. We also found that glipizide did not directly bind to either HMGIY or ANGPT1 proteins by ^1^H NMR, suggesting that HMGIY is not the direct target of glipizide (Supplemental Fig. 6). Interestingly, it has been reported by CL Zhang *et al*. that the cAMP sensor EPAC2 is a direct target of antidiabetic sulfonylurea drugs^36^. Furthermore, HMGIY knockout mice could develop a type II-like diabetic phenotype because of reduced-insulin receptors in the cell surface. Importantly, cAMP pathway can activate HMGIY and RBP4 and the activation of cAMP-HMGA1-RBP4 system has an important effect on the glucose homeostasis^37^. Thus, the accumulating evidence suggests that glipizide may target the cAMP/EPAC2/HMGIY/ANGPT1 signaling pathway in HUVECs.

Taken together, as one important finding of the present study, glipizide is identified capable of suppressing PC growth and metastasis in a TRAMP transgenic mouse model. Its therapeutic efficacy is dominantly associated with its capability of suppressing tumor-induced angiogenesis rather than PC cell proliferation. Moreover, regulating the HMGIY/ANGPT1 signaling is revealed as one of the dominant targets for the anti-angiogenic action of glipizide. Because angiogenesis is essential for tumor growth and metastasis, glipizide may be repurposed for an effective treatment of PC patients.

## Materials and Methods

### Ethics Statement

All animal experiments in this study were approved by the Medical Research Animal Ethics Committee of Guangdong Pharmaceutical University. All the protocols were approved by the Animal Care and Use Committee of the Guangdong Pharmaceutical University and performed in accordance with the approved guidelines.

### Reagents and cell lines

Glipizide was purchased from Sigma-Aldrich (cat. no. G117, Sigma-Aldrich, St Louis, MO, USA) and dissolved in DMSO to obtain 50 mg/mL stock solutions. Human PC-3, 22Rv1 and DU145 cell lines were presented as a gift by Assistant Professor Bo Wei (the Third Affiliated Hospital, Sun Yat-sen University).

### TRAMP transgenic mice

TRAMP transgenic mice with a C57BL/6 background were purchased from the Jackson Memorial Laboratory (Bar Harbor, Maine, USA). The genotypes of the TRAMP mice were determined by PCR using primers specific for TRAMP mice. The [C57BL/6 × FVB]F1 TRAMP mice were generated and maintained as previously reported^38^. The procedures were approved by the Animal Care and Use Committee of the Guangdong Pharmaceutical University.

### Experimental design

Sixteen-week-old [C57BL/6 × FVB]F1 TRAMP mice, which spontaneously develop PC, were introperitoneally treated every three days with DMSO or glipizide (5 mg/kg) for 8 weeks. The dose of the drug used by us was calculated according to the dose for patients (30 mg/patient) with the conversion formula of animal and human body weight: the dose of glipizide for every mouse = conversion factor (9.01)× the dose for every patient (0.55 mg/kg). After 8 weeks, the mice were sacrificed, and the GU tract, consisting of the bladder, prostate, seminal vesicles, and urethra, were removed and weighed. The DP, LP, VP, and AP lobes were dissected under an inverted microscope, with one lobe of each frozen pair laid in liquid nitrogen and the other in 10% formalin.

### Histopathology

Individual prostate lobes from the TRAMP mice were first fixed overnight in 10% formalin. Next, the fixed tissues were embedded in paraffin and sectioned at a thickness of 3 μM. The sections were stained with hematoxylin and eosin (H&E) according to standard protocols. Based on the TRAMP-specific grading scheme, as previously described by Suttie *et al*.^25^, the proliferative prostate lesions were assigned a semiquantitative score. The data from the prostate slides were collected using a double-blind protocol and evaluated independently by two observers.

### Evaluation of tumor metastasis

The lungs, livers, kidneys, spleens, brains and intestines of glipizide-treated or DMSO-treated mice were embedded in paraffin and sectioned serially. The sections were stained with H&E. To assess the number of metastatic and micro-metastatic foci and incidence of metastasis, the H&E-stained sections were subjected to microscopic examination by two experienced pathologists who were blinded to the source of the tissues. The metastatic foci in the serial sections were counted. The number of metastatic foci in every mouse was calculated with the following equation: number = the foci number of all the sections in every mouse/the section number.

### Rat aortic ring assay

The rat aortic ring assay was performed as previously described with minor modifications^39^. 7 to 9-week-old male Sprague-Dawley rats were obtained from experimental animal center of Guangdong Pharmaceutical University. The aorta, isolated from the Sprague-Dawley rats, was cut into 1–2 mm long rings and rinsed with ice-cold phosphate-buffered saline (PBS) three times. The rings then were placed into 100 μL Matrigel-coated 48 well plates and incubated at 37 °C in 5% CO_2_ for 30–45 minutes. EGM media (cat. no. CC-2935, LONZA, Walkersville, MD, USA) containing glipizide (25 μg/mL), ANGPT1 protein, or DMSO were added to the wells and incubated at 37 °C in 5% CO_2_ for 7 days. At the end of incubation, the aortic rings, formed the microvessel sprouting, were taken using the inverted microscope. The blood vessel outgrowth was quantified by counting the number of microvessels arising from the aorta rings^39^.

### *In vivo* Matrigel plug assay

Female Balb/c mice (5–6 weeks old) were purchased from Guangdong Medical Laboratory Animal Center (Guangzhou, China). Matrigel plug assays were performed as previously described^40^. The mice were subcutaneously injected with 500 μL of Matrigel (cat. no. 356230, BD Biosciences, Becton Dickinson, San Jose, CA) containing FGF-2 (150 ng/mL, cat. no. 3139-FB/CF, R&D systems, Minneapolis, MN, USA), heparin (60 units, Cisen pharmaceutical company, Shandong, China) and either glipizide (50 μg/mL) or DMSO. Seven days later, the mice were euthanized by cervical dislocation and the Matrigel plugs were isolated and removed. The images of Matrigel plugs were taken using Canon Power shot G10 digital camera. Angiogenesis was analyzed by measuring the amount of hemoglobin and immunofluorescence for CD31.

### Immunohistological and immunofluorescent staining

Immunohistological and immunofluorescent staining was used on 3-μm and 6-μm sections. Before the TRAMP mice were sacrificed, 100 mg/kg of 5-bromo-2′-deoxyuridine (BrdU; cat. no. B5002, Sigma) was injected into the transgenic mice. The sections were incubated with anti-CD31 (1:100 dilution, cat. no. sc-1506, Santa Cruz, CA, USA), anti-BrdU (1:100 dilution, cat. no. sc-32323, Santa Cruz, CA, USA) or ANGPT1 (cat. no. ab8451, Abcam, Cambridge, CB, UK) antibodies overnight at 4 °C. HRP**-**conjugated or DyLight 488-conjugated or DyLight 555-conjugated secondary antibodies were then added to the sections and stained with DAB and hematoxylin or 4′-6-diamidino-2-phenylindole (DAPI). For the microvessel density and BrdU quantitation, the CD31^+^ vessels were counted in a 200× field^41^, and the number of BrdU^+^ cells was counted in a 400× field and expressed as a percentage of the total cells per field^42^. The images of immunofluorescent staining were quantified using an image analysis program IPP 6.0 (Image Pro-Plus, version 6.0, Media Cybernetics).

### MTT assay

An MTT assay was performed to assess the effect of glipizide on PC cell proliferation. Briefly, the cells were added to 96-well plates and then treated with glipizide (50–200 μM) and DMSO for 48–72 h. Next, the MTT reagent was introduced to each well, and the supernatants were removed 4 h later. A total of 150 μL DMSO (Sigma-Aldrich) was used to dissolve the resultant formazan crystals. The absorption was read at 570 nm using a spectrophotometer.

### Flow cytometry

To further determine whether glipizide influences tumor cell proliferation, flow cytometry analysis was performed as previously described^43^. The cells were treated with 200 μM glipizide or DMSO for 48 h. Forty-eight hours later, the cells were resuspended in PBS and fixed with ice-cold 70% ethanol at 4 °C overnight. Subsequently, the cells were stained with PI solution at 4 °C for 30 min after the cells were washed twice with PBS. Then the PC cells (1 × 10^6^/mL) were subjected to a fluorescence-activated cell sorting flow cytometer (Cytomics™ FC 500; Beckman Coulter, Miami, FL, USA) and the percentages of cells in different phases of the cell cycle were quantified with the Modfit software.

### Quantitative real-time PCR

Total RNA was isolated from glipizide- or DMSO-treated HUVECs. The qRT-PCR array kit was then used to analyze the angiogenesis-associated gene. All qRT-PCR assays were run on an ABI PRISM 7000HT Sequence Detection System (Applied Biosystems).

### Western blotting

The protein was extracted after HUVECs were treated with glipizide (5 μg/mL) and DMSO or transfected with siRNAs. The total proteins were separated and transferred onto nitrocellulose membranes. The membranes were incubated with ANGPT1 antibodies (Abcam) at 4 °C overnight and then with horseradish peroxidase-coupled IgG. The Odyssey Infrared Imager (LICOR Bioscience, Lincoln, NE, USA) was used to detect the bands.

### Statistical analyses

GraphPad Prism 5 software package (GraphPad Software, CA) was used to analyze the data and draw the statistical charts. After satisfying the prerequisites (independence and normal distribution), a two-tailed Student’s t-test was used to confirm whether certain responses, which included IHC results, the GU tract weights, microscopic lesion scores and distributions, and MTT results, were affected by glipizide administration. Differences between groups were considered significant at *p* < *0.05*.

## Additional Information

**How to cite this article**: Qi, C. *et al*. Glipizide suppresses prostate cancer progression in the TRAMP model by inhibiting angiogenesis. *Sci. Rep.* **6**, 27819; doi: 10.1038/srep27819 (2016).

## Supplementary Material

Supplementary Information
