# Supplementary material for: Glipizide suppresses prostate cancer progression in the TRAMP model by inhibiting angiogenesis
Source: Sci Rep. 2016 Jun 13;6:27819. doi: 10.1038/srep27819 (PMC4904209; doi:10.1038/srep27819)
Supplement: Supplementary Information [file srep27819-s1.doc]

**Glipizide suppresses prostate cancer progression in the TRAMP model by inhibiting angiogenesis**

Cuiling Qi^1^,*, Bin Li^1^,*, Yang Yang^1^, Yongxia Yang^1^, Jialin Li^1^, Qin Zhou^1^, Yinxin Wen^1^, Cuiling Zeng^1^, Lingyun Zheng^1^, Qianqian Zhang^1^, Jiangchao Li^1^, Xiaodong He^1^, Jia Zhou^3^, Chunkui Shao^2^,**, Lijing Wang^1^,**

^1^Vascular Biology Research Institute, Guangdong Pharmaceutical University, Guangzhou 510006, China

^2^Department of Pathology, the Third Affiliated Hospital, Sun Yat-sen University, Guangzhou, Guangdong 510630, China

^3^Chemical Biology Program, Department of Pharmacology and Toxicology, University of Texas Medical Branch, Galveston, TX 77555, United States

*These authors contributed equally to this work.

*These authors contributed equally to this work.

**Correspondence and requests for materials should be addressed to Lijing Wang ([wanglijing62@163.com](mailto:wanglijing62@163.com)) and Chunkui Shao (chunkuishao2011@163.com).

**Supporting Information**

**Table of Contents: 1. Method, 2. Supplemental Figures**

**1. Method: The ^1^H NMR.** Page 3

**2. Supplemental Figures**

**Figure S1.** Representative H&E images of VP, LP and AP tissues from TRAMP mice treated with DMSO and glipizide. Page 4

**Figure S2.** Representative immunohistological images of VP, LP and AP tissues from TRAMP mice treated with DMSO and glipizide. Page 5

**Figure S3.** The effect of glipizide on body weights and blood lipid levels of the TRAMP mice. Page 6

**Figure S4.** The effect of glipizide on the transgene expression. Page 7

**Figure S5.** The effect of glipizide on ANGPT1-receptor and angiogenesis. Page 8

**Figure S6.** The 1H NMR spectra (δ0.5-8.0). Page 9

**Table S1.** The effect of glipizide on the blood glucose in TRAMP mice. Page 10

**Method**

**The ^1^H NMR**

The ^1^H NMR spectra of all samples were collected at 298 K on a Bruker Avance III 500 MHz spectrometer. The ^1^H NMR spectra were recorded using the ZGPR pulse sequence. Three hundred and twenty transients were collected with 64k data points using a spectral width of 10 kHz with a relaxation delay of 3 seconds. The exponential function corresponding to a line broadening factor of 0.3 Hz was applied to all acquired free induction decays (FIDs) before Fourier transformation.

**Supplemental Figures**

**Figure S1.** Representative H&E images of VP, LP and AP tissues from TRAMP mice treated with DMSO and glipizide. To determine the histologic appearance in the PC of TRAMP mice treated with glipizide or DMSO, H&E staining was performed and the images were taken. Bar, 20 μm.


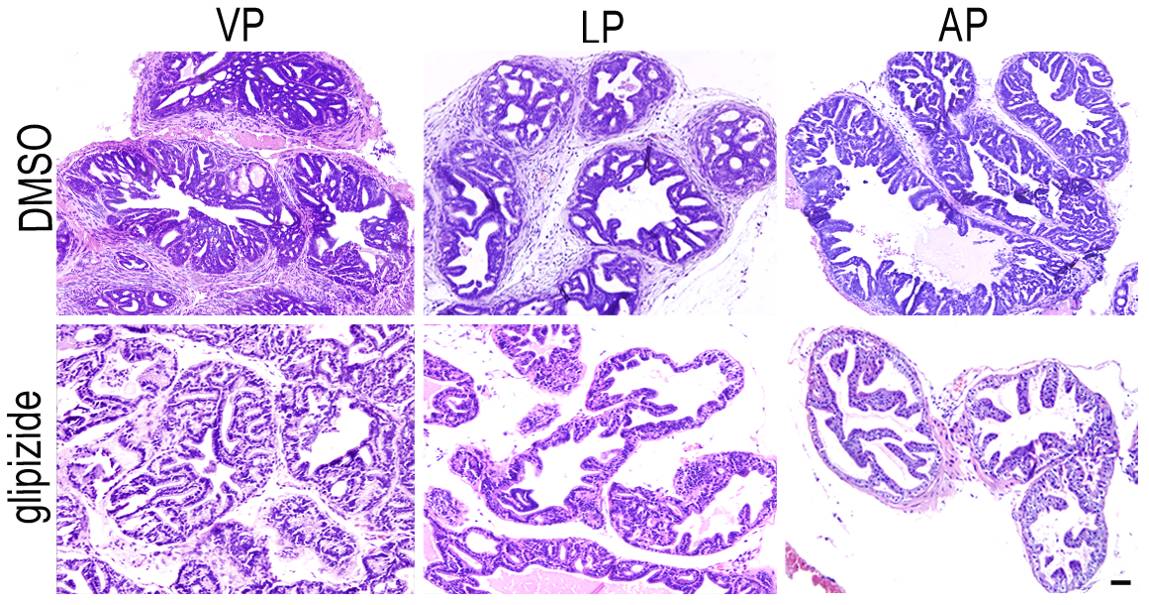


**Figure S2.** Representative immunohistological images of VP, LP and AP tissues from TRAMP mice treated with DMSO and glipizide. (A) Immunohistological staining for blood vessel with CD31 was performed in the VP, LP and AP lobes of the prostates and the images were taken. (B) Immunohistological staining against BrdU was performed on VP, LP and AP lobes of the prostates and the images were taken. Bar, 20 μm.


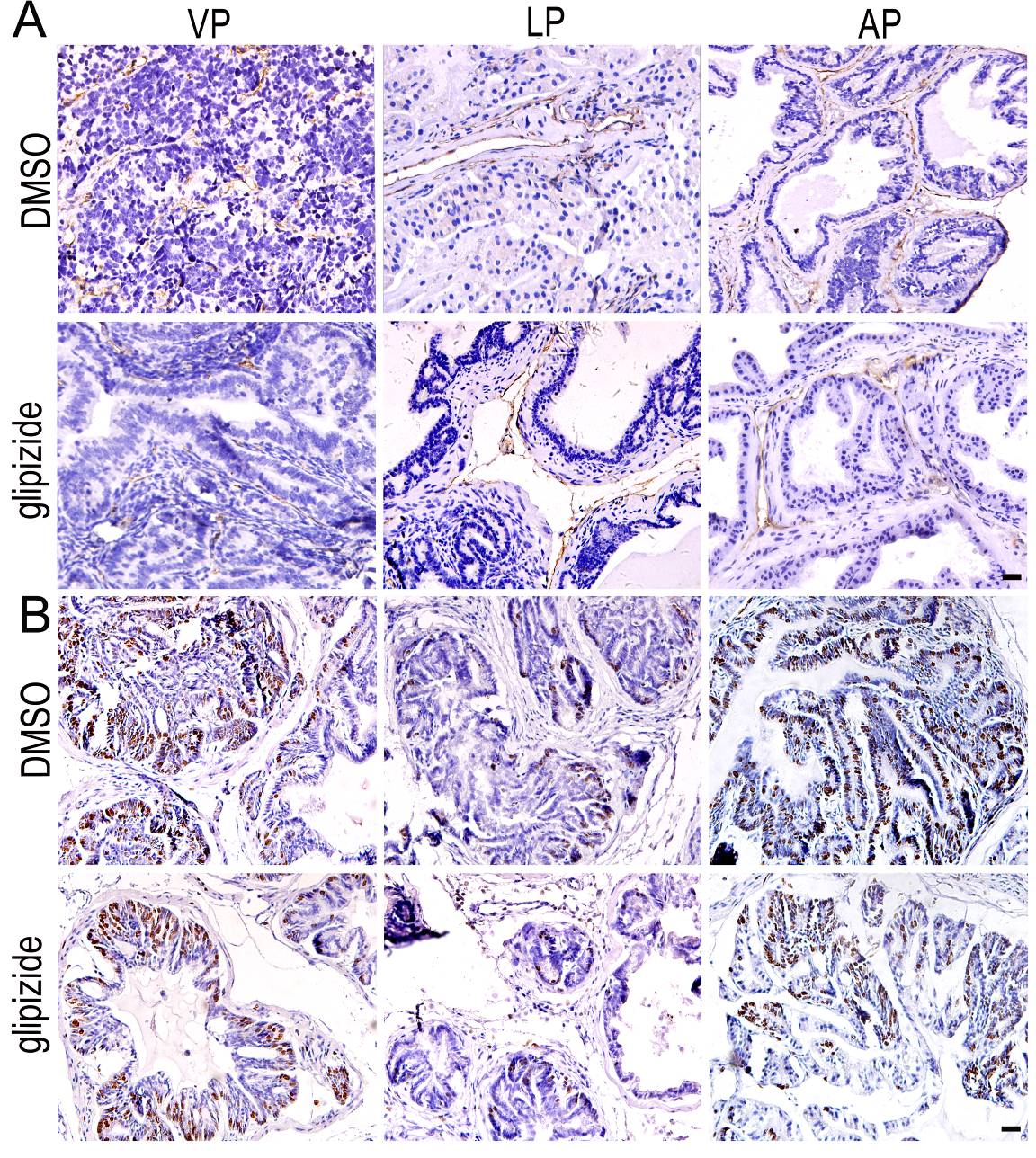


**Figure S3.** The effects of glipizide on body weights and blood lipid levels of the TRAMP mice. The body weights of the TRAMP mice were measured weekly. The results demonstrated that glipizide displayed no significant effects on body weights (A) and blood lipid levels (B-D).

**
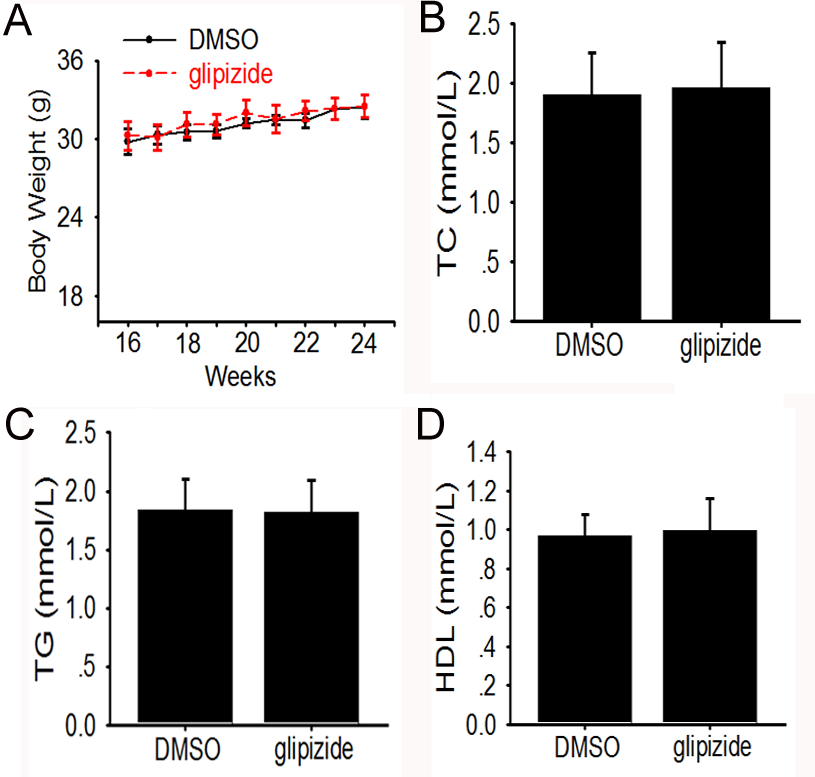
**

**Figure S4.** The effect of glipizide on the transgene expression. Immunohistological staining against T-antigen was performed on the AP lobes of the prostates and the images were taken. The IHC results showed that there were no significant effects on transgene expression in glipizide-treated mice as compared with the DMSO-treated mice. Bar, 20 μm.

**
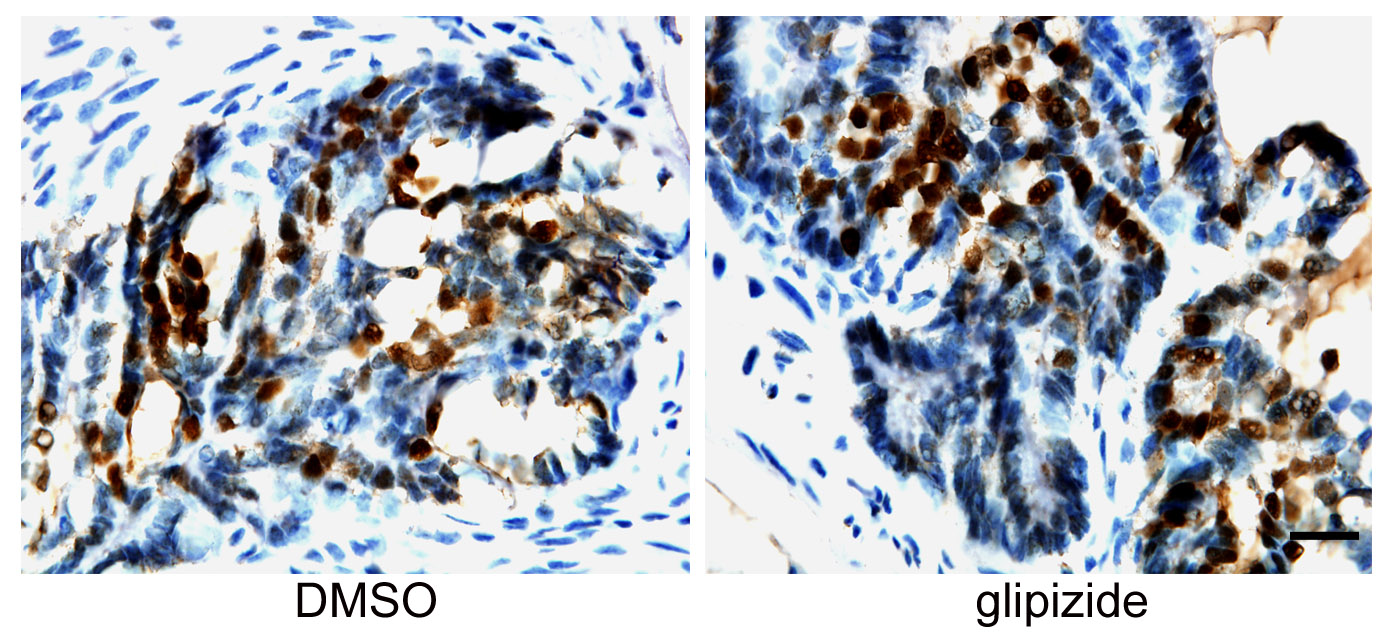
**

**Figure S5.** The effect of glipizide on ANGPT1-receptor and angiogenesis. (A) The aorta was isolated from the Sprague-Dawley rats and cut into 1-2 mm long rings. The aortic rings from the Sprague-Dawley rats were placed into matrigel-coated plate and treated with DMSO, glipizide, ANGPT1 or ANGPT1 and glipizide. Representative photograghs of sprouts from aortic rings were taken. (B) Microvessel outgrowth was quantified. (C) Glipizide significantly up-regulated the Tie2 and p-Tie2 protein expression in HUVECs. (D) The HUVEC tube formation was not affected by exogenous ANGPT1 after HMGIY was silenced. Bar, 20 μm. * *p* < 0.05, ** *p* < 0.01, *** *p* < 0.001.


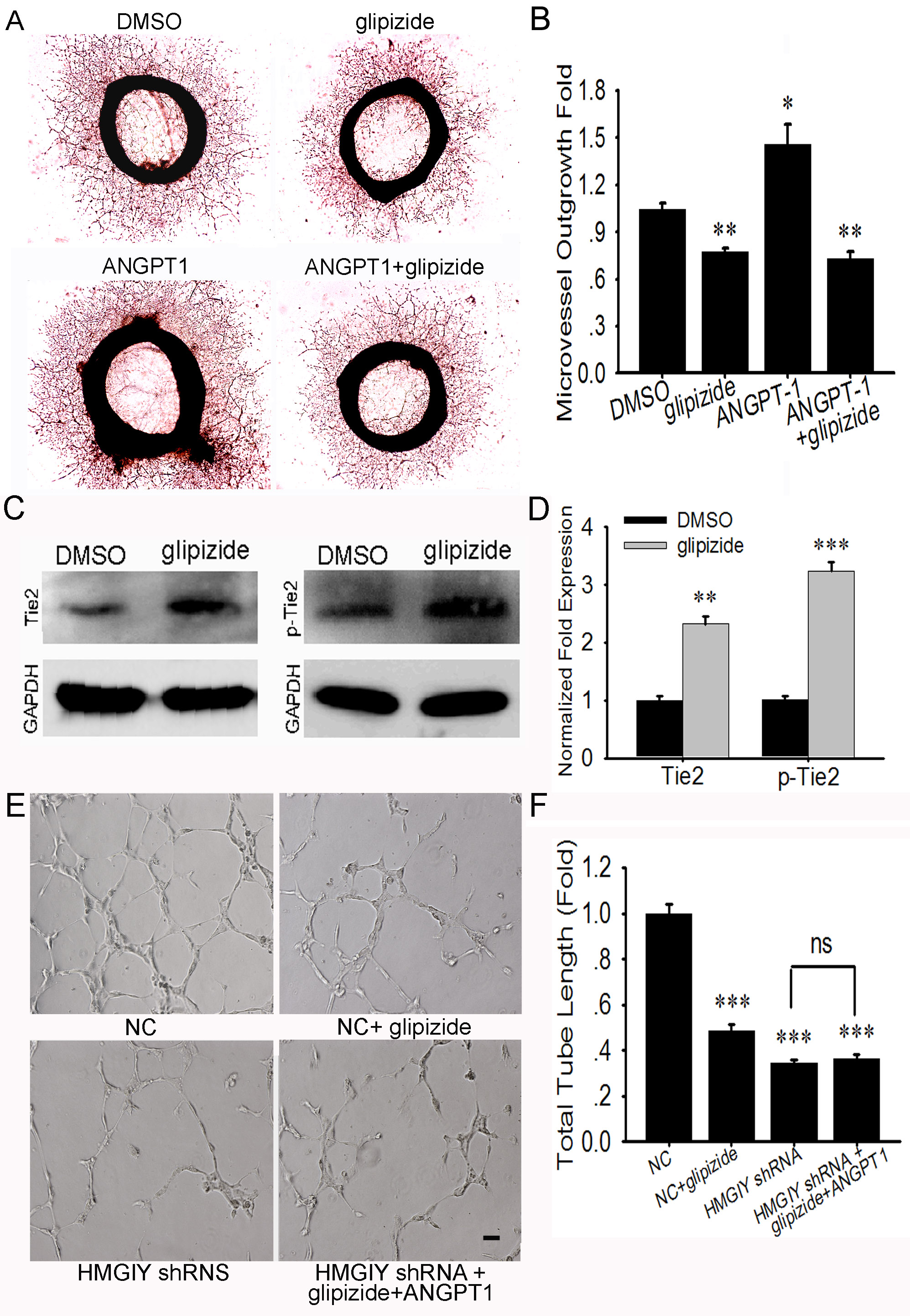


**Figure S6.** The ^1^H NMR spectra (δ0.5-8.0). The 1H NMR spectra of all samples were given in the figures, in which the three labeled singles at δ1.05-1.1, δ1.55-1.65 and δ2.63 were assigned to glipizide. If glipizide binds to HMGIY or ANGPT1 proteins, the chemical shift and peak shape of glipizide are supposed to change because of the space combination. Compared with the spectra of glipizide (B), the chemical shift and peak shape of glipizide + ANGPT1 were not changed, demonstrating that there was no interaction between glipizide (B) and ANGPT1 (C and D), in a similar fashion to that between glipizide (B) and HMGIY (E and F).


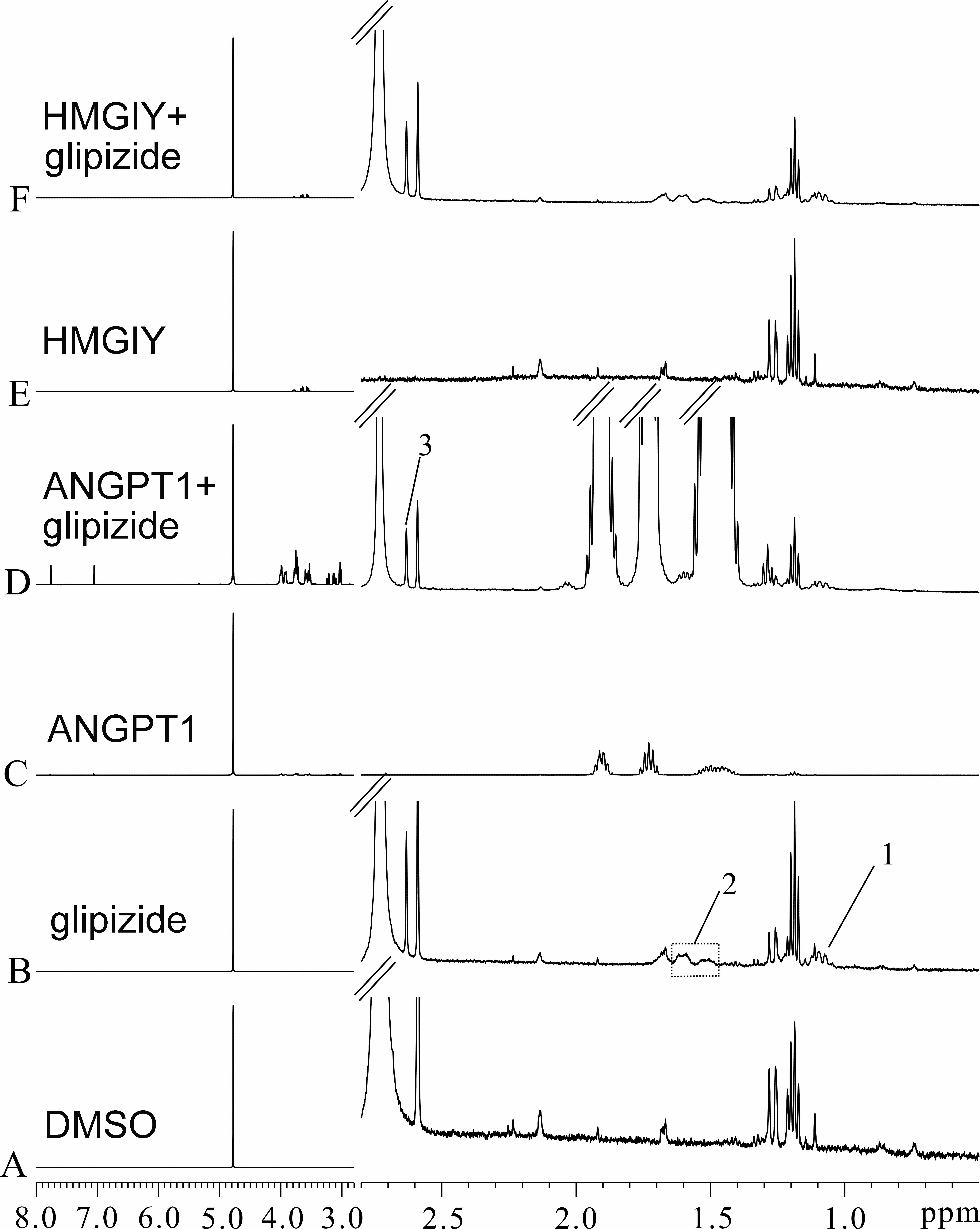


**Table S1.** Glipizide decreased the blood glucose of TRAMP mice. The postprandial blood glucose levels of the TRAMP mice treated with glipizide were significantly reduced in 0.5 h and 12 h. ** *p* < 0.01.

| Groups | Postprandial blood glucose | | |
| --- | --- | --- | --- |
|  | 0 h | 0.5 h | 12 h |
| DMSO | 8.83 ± 0.26 | 8.67 ± 0.35 | 9.12 ± 0.28 |
| glipizide | 8.95 ± 0.41 | 5.44 ± 0.33** | 6.34 ± 0.25** |
